# INVITATION TO THE 8^TH^ GHA CARDIOVASCULAR CONFERENCE

**Published:** 2010

**Authors:** Hajar Ahmed Albinali

**Affiliations:** *GHA President*

The field of cardiovascular medicine and its sub-specialties are advancing at a rapid pace. It is becoming hard for general cardiologists, let alone internists and general practitioners, to keep up with the changes and progress in this field.

The Gulf Heart Association (GHA) is the main cardiovascular society in seven Arab countries forming the Gulf Cooperation Council (GCC). They are: Bahrain, Kuwait, Kingdom of Saudi Arabia, Qatar, Oman, UAE, and Yemen. The GHA with its specialized members and international visitors aim at spreading knowledge in the prevention and management of cardiovascular disease. It is a must for all practicing cardiovascular specialists to be familiar with such new knowledge and to incorporate these new strategies in their practice.

The main goal of the GHA is to improve cardiovascular care in the GCC states. One of the important ways to achieve this goal is to gain new knowledge and master new skills through attending scientific conferences such as this 8th annual GHA meeting. We hope that with such a conference the GHA will help raise the quality of health care in general and cardiovascular health in particular.

To fulfill the objectives of the GHA, improve the quality and raise the standard of cardiac care in the GCC, the GHA since its creation in 2002 has conducted and will continue to pursue the following activities:

- Conduct scientific conferences and symposia.
- Carry on scientific research on cardiovascular diseases. The GHA acute coronary syndrome registry is well known as the only successful collaborative multicenter medical study in the Gulf, the results of which are published in several leading international cardiovascular journals. This study had a good impact on our practice. There are at this time other studies started by the GHA such as atrial fibrillation.
- Publish professional periodicals and information hoping to establish criteria for GCC cardiovascular specialists to meet high standards of competence and expertise with:
  - Pocket guidelines on cardiovascular management. These are published by the society such as Acute Coronary Syndrome, ST-Elevation Myocardial Infarction and Venous Thrombo-Embolism.
  - Heart Views as the GHA official journal. Website: (http://www.gulfheart.org/)
- Create professional, educational and social ties among members of the GHA.
- Create links and cooperation with international medical institutions and professional societies such as the ESC and ACC.
- The GHA seeks to suggest new laws to be adopted by the GCC countries for the prevention of cardiac disease and advance the care of patients with heart disease.
- The GHA is a torch for science and cooperation among us for the progress of our profession and to provide advanced care to the citizens of our beloved GCC states.

Each GCC country takes its turn in hosting the annual GHA conferences. The first conference was held in Doha, Qatar in 2002. Now after all the GCC countries had an opportunity to host the GHA conference once, it is coming back to Doha as the 8th GHA conference in April 8-10, 2010 to start a new circle of rotation. The GHA is planning to make this coming conference a model conference and a leap forward in its topics, organization, participation and attendance. We are pleased that both the American College of Cardiology and the European Society of Cardiology will have joint sessions with our GHA in this meeting. Over one thousand participants have already registered online for attending the workshops. Besides the GHA members and other cardiologists in the GCC countries, several regional cardiac societies’ presidents and representatives from Jordan, Egypt, Syria, Lebanon and Tunisia among others, will join us in Doha in April.

I would like to take this opportunity to invite all health care professionals to attend, help and participate in our GHA activates.

**Teach us and learn from us.**

